# Modalities and dosage of exercise interventions for improving adolescent mental health: a systematic review and meta-analysis

**DOI:** 10.3389/fpsyg.2026.1986368

**Published:** 2026-10-28

**Authors:** Wengao Liao, Wenlu Yu, Yuanji Zhao, Guiqing Li, Zonglin Ma, Jie Xie

**Affiliations:** 1 School of Physical Education of Wuhan Sports University, Wuhan, Hubei, China; 2 College of Physical Education of Chengdu University, Chengdu, Sichuan, China; 3 College of Physical Education of Civil Aviation Flight University of China, Deyang, Sichuan, China; 4 School of Physical Education, Tianfu College, Southwestern University of Finance and Economics, Mianyang, Sichuan, China

**Keywords:** adolescent, exercise, mental health, network meta-analysis, public

## Abstract

**Objective:**

This study aims to: (1) update existing evidence on exercise interventions improving adolescent mental health; (2) exploring potentially effective models and dosages of exercise interventions to improve adolescents’ mental health; (3) provide evidence-based medical insights to inform future research and non-clinical treatments for mental disorders.

**Methods:**

We searched six databases—Web of Science, PubMed, Embase, The Cochrane Library, SportDiscus, and Scopus—for randomized controlled trials (RCTs) on exercise interventions for adolescent mental health published since the databases were established. We used the Rob 2.0 risk of bias tool to assess the methodological quality of the included studies, and then performed conventional meta-analyses, network meta-analyses, subgroup analyses, and sensitivity analyses using R software.

**Results:**

A total of 17 randomized controlled trials were included. The meta-analysis results showed that, compared with the control group, exercise intervention had a significant positive effect on improving adolescents’ depression (g = −0.34, *p* = 0.0109), anxiety (g = −0.45, *p* < 0.0001), and stress (g = −0.49, *p* < 0.0001). Subgroup analysis and sensitivity analysis results indicate that the high heterogeneity among groups is likely due to an imbalance in the number of studies and sample sizes across subgroups. Based on the results of a network meta-analysis and SUCRA probability ranking, adolescents may derive potential benefits from low-intensity TCC exercises performed less than three times per week, with each session lasting more than 60 min; anxiety symptoms may benefit from moderate-to-high-intensity HIIT sessions lasting more than 60 min per session, performed fewer than three times per week; and low-intensity yoga sessions lasting 30–60 min per session, performed fewer than three times per week, may help alleviate stress symptoms in adolescents. Due to the limited number of studies included, the Egger’s test was not statistically significant, and publication bias cannot be ruled out; therefore, the results should be interpreted with caution.

**Conclusion:**

Exercise intervention, as an alternative non-pharmacological treatment approach, plays a positive role in improving adolescents’ mental health. The exercise regimens and dosages recommended in this study are based on a synthesis of the existing evidence; further research is needed to explore the effects of interventions involving different variables.

**Systematic review registration:**

The study has been registered with the International Prospective Systematic Reviews Register (PROSPERO) (CRD420251087193).

## 1 Introduction

The current mental health status of adolescents worldwide remains concerning and has become an increasingly serious global public health issue (Scott et al., 2025). Depression is a mental disorder characterized by high prevalence, a high clinical remission rate, low treatment uptake, and a high relapse rate (Pahlavani, 2024). According to World Health Organization data, approximately 280 million people worldwide suffer from depression, while hundreds of millions are affected by anxiety disorders and excessive stress. In Southeast Asia, the incidence of depression among adolescents and suicidal ideation has been increasing significantly year by year (Fine et al., 2025). Developed countries such as the United States are experiencing similar situations, which have increased the burden on national mental health systems (Olfson et al., 2024). In response to this pressing issue, some countries and medical institutions have proactively introduced policy documents and recommendations concerning the prevention and treatment of adolescent mental health (Patel et al., 2023; Roche et al., 2024). This underscores the urgent need for effective interventions addressing adolescent mental health issues. While various approaches exist to improve mental health, only a small proportion of individuals receive clinical treatment for specific psychological disorders. However, clinical psychological therapy carries high costs and has not achieved the expected remission rates, with only about 50% of cases showing improvement (Cohan et al., 2022). Furthermore, antidepressants and similar medications may cause side effects. Research indicates that due to medication side effects and feelings of shame, the majority of adolescents with depression refuse further treatment after their initial psychological and pharmacological interventions (Edbrooke-Childs et al., 2022).

Current research confirms that physical activity effectively improves mental health among adolescents. Evidence indicates that children and adolescents who engage in 60 min or more of moderate to vigorous physical activity daily reap substantial benefits across multiple domains of health and fitness (Chen et al., 2020). This lifestyle medicine has garnered widespread attention for offering low-cost, convenient, and readily accepted treatment methods, emerging as a promising alternative therapy (Blumenthal and Rozanski, 2023; Zhao et al., 2024). Most current research focuses on the impact of exercise interventions on specific aspects of adolescent mental health, such as depression, negative emotions, and perceived well-being (Dings and Spinath, 2024; Linhares et al., 2024; Rong et al., 2024), but the impact of different types and modes of exercise on adolescent mental health remains a topic for further discussion, and common mental health exercise interventions need to be more widely adopted. Therefore, to better serve adolescent mental health prevention and treatment efforts, this study evaluates the overall impact of exercise intervention on adolescent mental health across three dimensions—depression, anxiety, and excessive stress—to explore the optimal exercise patterns and dosages for improving adolescent mental health. This research aims to provide reference information for non-clinical treatment approaches to mental health disorders.

## 2 Methods

### 2.1 Registration of systematic review protocol

This study adheres to the preferred reporting items outlined in the 2020 Preferred Reporting Items for Systematic Reviews and Meta-Analyses (PRISMA) guidelines (Liberati et al., 2009; Page et al., 2021) and ensured the reasonableness and accuracy of the report. The study has been registered with the International Prospective Systematic Reviews Register (PROSPERO) (CRD420251087193).

### 2.2 Search strategy

Before the search, one of the authors (LWG) conducted a review of the research topic within the Web of Science retrieval system. A retrieval strategy was developed based on specialized terminology commonly used in adolescent mental health research, frequently employed interventions, and commonly utilized outcome measures to maximize the retrieval of studies relevant to the research topic (Xu et al., 2025).

On December 28, 2025, a Boolean search using subject headings combined with free-text terms was conducted in five databases—Web of Science (Core Collection), PubMed, Embase, The Cochrane Library, SportDiscus, and Scopus. The search terms were structured around six conceptual groups: “terms related to “adolescent/teenager/youth/juveniles,” terms related to “mental health/psychological health/psychological well-being,” terms related to “despondent/clinical depression/depression/depressive disorder/depressive illness,” terms related to “neurosis/nephropathy/anxiety disorder/anxiety,” terms related to “stress/strain/stress symptoms,” and “randomized controlled trials,” Within each concept group, synonyms were linked using “OR,” while the groups themselves were linked using “AND.” Detailed search strategies for each database are provided in Appendix 1. In addition, the database search results were supplemented and refined using ResearchGate’s literature tracking feature and the “snowball” method (Yin et al., 2025).

### 2.3 Study selection and eligibility criteria

One author (LWG) identified and screened the literature. Subsequently, two reviewers (YWL and ZYJ) independently reviewed the full texts in strict accordance with the exclusion and inclusion criteria established based on the PICOS framework. Inter-rater reliability was assessed using Cohen’s kappa coefficient, which showed a consistency of 0.85 between the two reviewers (YWL and ZYJ) in study selection, indicating that the study selection results are reliable(McHugh, 2012). In cases where it was unclear whether a study should be excluded, discrepancies were resolved through discussion between the two reviewers. The entire process was conducted using EndNote X20 software. The eligibility criteria for the articles are provided in Appendix 2.

### 2.4 Data extraction

Two reviewers (WL and WY) extracted data using a standardized Excel template designed by the team. The extracted information included: (1) basic study information: first author’s name and year of publication; (2) participant characteristics: number of participants, gender, and age; (3) intervention characteristics: exercise intensity, exercise cycle, exercise duration, exercise frequency, and exercise type; and relevant statistical data on outcomes related to adolescent depression, anxiety, and stress at the end of the intervention. In cases where data were missing from the literature, the corresponding authors of the relevant studies were contacted to request the information. If no response was received to the initial email, a follow-up email was sent 24 h later. If no response was received within 1 week, the study was excluded from the analysis.

### 2.5 Quality evaluation and risk of bias evaluation

Assessing the risk of bias using the Cochrane Collaboration’s Rob 2.0 tool (Sterne et al., 2019). This tool assesses the risk of bias in included studies across five modules: bias arising from randomization processes, bias from deviation from the intended intervention, bias from missing outcome data, bias from outcome measurement, and bias from selective reporting of results. Based on the assessment results, the Rob 2.0 tool generates a risk of bias plot and a summary of risk of bias. In addition, the Physical Therapy Evidence Database (PEDro) scale was used to assess the methodological quality and reporting completeness of the included studies (de Morton, 2009; Ludyga et al., 2016). This scale evaluates the included studies based on the following criteria: (1) whether the inclusion criteria for participants are clearly specified; (2) whether participants were randomly assigned to groups; (3) whether the allocation method was concealed; (4) whether the comparability of groups was assessed at baseline; (5) whether participants were blinded; (6) whether therapists were blinded; (7) whether the raters of measurement outcomes were blinded; (8) whether the primary outcome was measured in at least 85% of participants; (9) whether participants received the treatment or control condition as assigned; (10) whether the statistical analysis was appropriate; (11) whether mean and standard deviation values for the primary outcome were provided. The PEDro scale ranges from 0 to 10 points, with scores of ≤4 indicating low quality, 5–7 indicating moderate quality, and >7 indicating high quality. ROB 2.0 is used to assess the risk of major bias, while PEDro is used to supplement the assessment of information related to the quality of reporting and external validity of exercise intervention studies. The combined use of the ROB 2.0 tool and the PEDro scale allows for a systematic assessment of bias risk alongside an evaluation of the specific methodological characteristics of exercise intervention trials, thereby providing a more comprehensive reflection of the quality of the included studies. Specifically, the overall bias risk assessment from ROB 2.0 serves as the primary basis for evidence quality grading, while the PEDro score is used as supplementary information to describe the methodological quality of the studies and the completeness of the intervention reporting.

### 2.6 Statistical analyses

This study used R software (version 4.5.1) to conduct a pairwise meta-analysis. Since different studies used different psychological assessment tools, we employed Hedges’ g as the standardized effect size to assess the standardized mean difference (SMD) and standardized the scale directions. The pooled effect size was calculated based on the post-intervention mean difference (Wang et al., 2022), That is, comparing the outcome scores between the intervention group and the control group at the end of the intervention. Hedge’s g was classified as trivial (<0.2), small (0.2–0.5), medium (>0.5–0.8), and large (>0.8). For studies lacking a standard deviation (SD) but reporting 95% confidence intervals (da Silva et al., 2025; Smith et al., 2025; Zheng et al., 2021), data were converted to obtain SD values using the sample size (N) and the upper and lower limits of the 95% confidence interval for the standardized mean difference. When the sample size was ≥100, we reconstructed the standard deviation using the formula SD = $\sqrt{N}$ × (UCI - LCI)/3.92; when the sample size was ≤60, we used the formula SD = $\sqrt{N}$ ×(UCI - LCI)/(2 × t) to ensure that the results of each study were standardized. Given the heterogeneity resulting from differences in demographic and other factors, this meta-analysis employed a random-effects model throughout (Yang et al., 2025). The *I^2^* statistic is used to assess heterogeneity among studies, with values ranging from 0 to 100%. When *I^2^* ≤ 50%, the level of heterogeneity among studies is negligible. When *I^2^* > 50%, subgroup analysis and sensitivity analysis are conducted to explore the sources of heterogeneity.

We also used R software (version 4.5.1) to conduct a network meta-analysis. We employed the “gemtc” package and a Bayesian random-effects model to account for expected heterogeneity across studies. Bayesian NMA were implemented using the Markov chain Monte Carlo algorithm with 4 chains and 50,000 interactions per chain (burn-in period of 20,000). Trace plots were used for convergence assessment. If the transitivity assumption cannot be directly verified through statistical testing, it is assessed based on clinical and methodological soundness. First, we visualized the relationships between direct and indirect comparisons of different interventions by plotting a network evidence graph. When the network formed a closed loop, we assessed inconsistencies using a node-splitting method and reported the corresponding *p*-values (Rouse et al., 2017). If *p* > 0.05 and the 95% confidence interval includes 0, consistency within that ring is considered acceptable; otherwise, inconsistency is deemed to exist. On the other hand, best-probability ranking uses the area under the cumulative ranking curve (SUCRA) for ranking. SUCRA ranges from 0 to 100%; the higher the SUCRA value, the higher the intervention’s ranking in the network, indicating that the intervention has a greater improvement effect (Xie et al., 2026).

## 3 Results

### 3.1 Search results

A database search yielded a total of 2,857 articles. After removing duplicates, 1,173 articles remained. After reviewing the titles and abstracts, 1,095 articles unrelated to the research topic were excluded, leaving 78 articles for eligibility screening. A further 60 articles were excluded due to unreasonable interventions, flawed study designs, missing outcome measures, missing experimental data, or unavailability of full-text articles. Ultimately, 10 articles identified through the database search were included in the meta-analysis. Additionally, 3 studies were sourced from other channels. After rigorous screening based on the inclusion criteria, a total of 13 studies were included in the meta-analysis (Appendix 3).

### 3.2 Study characteristics

Since a single study may include multiple randomized controlled trials, a total of 17 randomized controlled trials from 13 studies were included in the meta-analysis following a rigorous eligibility review, involving a total of 4,220 participants, including 2,134 in the intervention group and 2,086 in the control group. Exercise intensity was predominantly low; the duration of the exercise programs ranged from 2 to 17 weeks; the duration of a single exercise session ranged from 20 to 90 min; and the frequency of exercise per week ranged from 1 to 5 times. The types of exercise primarily consisted of traditional exercises (aerobic, resistance, and strength training) and mind–body exercises (yoga, tai chi). According to the PEDro score, a total of 8 trials were rated as high quality (Smith et al., 2025; Zheng et al., 2021; Philippot et al., 2022; Liu et al., 2025; Zhang et al., 2018; Gu et al., 2024). Five studies were rated as moderate quality (da Silva et al., 2025; Butzer et al., 2016; Ranjani et al., 2023; Wunram et al., 2018; Parajuli et al., 2022). Four studies were rated as low quality (Wu et al., 2025; Li and Zhou, 2025). For detailed information regarding the experimental samples and intervention characteristics, (see Appendix 4).

### 3.3 Risk of bias and quality assessment

The results shows that all studies reported random allocation of participants; however, because exercise intervention is a complex treatment involving behavioral change—and is characterized by visibility and active participation—it is extremely difficult to achieve double-blind conditions in these studies. Eight studies used a single-blind design or did not conceal allocation; Regarding bias arising from deviation from the intended intervention, two studies had some risk of bias in intervention allocation and adherence that did not affect the outcomes; therefore, they were rated as “Some concerns”; None of the trials presented a clear risk of bias regarding missing outcome data; regarding outcome measurement and selective reporting, only a few trials exhibited potential measurement and reporting biases that likely did not affect the outcome variables; Overall, the risk of bias arising from the randomization process was the key factor affecting the overall quality of the trials. The risk of bias plots and summary risk plots for the included studies are shown in Appendix 5, Figure 2.

The PEDro score results show that the included studies had an average PEDro score of 6.8, indicating that the overall methodological quality of the included studies was above average. The PEDro methodological quality assessment forms for the included studies are presented in Appendix 5, Table 4.

### 3.4 Pairwise meta-analysis results

A meta-analysis of 17 randomized controlled trials showed that, Compared with the control group, the exercise intervention had a significant effect on adolescents’ depression (k = 10, g = −0.34, 95% CI: −0.58 to −0.11, *I*^2^ = 58.0%, *p* = 0.0109), anxiety (k = 10, g = −0.45, 95% CI: −0.71 to −0.19, *I*^2^ = 84.7%, *p* < 0.0001), and stress (k = 6, g = −0.49, 95% CI: −0.67 to −0.30, *I*^2^ = 24.5%, *p* < 0.0001) among adolescents. However, there was high heterogeneity among the studies; further subgroup and sensitivity analyses should be conducted to investigate the sources of this heterogeneity. See Appendix 6, Figure 3 for summary effect size plots of the various outcome measures.

To investigate whether the heterogeneity among the studies was caused by a single publication, a sensitivity analysis using the stepwise exclusion method was conducted (Howard et al., 2020; Mathur and VanderWeele, 2020). After sequentially excluding individual studies, the pooled effect estimates for all studies remained within the 95% confidence interval, indicating that the heterogeneity was not attributable to any single study, suggesting that the findings are robust. The results of the sensitivity analysis based on the leave-one-out method are shown in Appendix 6, Figure 4.

To further investigate the sources of heterogeneity, based on the characteristics of the interventions included in the study and the FITT principles established by authoritative bodies such as the American College of Sports Medicine (Cuijpers et al., 2021; Sweegers et al., 2018). The four factors—frequency, intensity, duration, and type—are interdependent and interact with one another, collectively forming a multidimensional exercise dose. Therefore, this study conducted subgroup analyses on four potential moderating variables—exercise frequency, exercise intensity, exercise duration, and exercise type—that may contribute to high heterogeneity. To ensure the validity of the subgroup analysis results, exercise frequency was divided into two subgroups (<3 times and ≥3 times), exercise intensity into two subgroups (low intensity and moderate-to-high intensity), exercise duration into three subgroups (≤30 min, 30–60 min, and ≥60 min), and exercise type into two subgroups (conventional exercise intervention and mind–body exercise intervention) for analysis.

The results of the subgroup analysis of the exercise intervention on depressive symptoms in adolescents are shown in Appendix 6, Figure 5, showed that sessions of ≤30 min (k = 2, g = −0.24, 95% CI: −0.65 to 0.18, *I^2^* = 48.3%, *p* = 0.1644) and 30–60 min (k = 3, g = −0.15, 95% CI: −0.36 to 0.07, *I^2^* = 0.0%, *p* = 0.6627) showed significant heterogeneity, indicating that these two subgroups were the primary sources of high intra-group heterogeneity; The results of the subgroup analysis of the exercise intervention on anxiety symptoms in adolescents are shown in Appendix 6, Figure 6, showed that there were significant changes in heterogeneity between the 30–60-min (k = 1, g = −0.20, 95% CI: −0.48 to 0.08, *I^2^* = NA, P=NA) and mind–body exercise (k = 2, g = −0.23, 95% CI: −0.46 to 0.00, *I^2^* = 0.0%, *p* = 0.6933) showed significant heterogeneity, indicating that these two subgroups are the primary contributors to the high heterogeneity. Overall, given the limited number of studies included for certain intervention points and subgroups, it is difficult to achieve sufficient statistical power to detect interactions among subgroups, and clinical and methodological heterogeneity across studies may be magnified; therefore, the results of subgroup analyses should not be used as a direct basis for clinical decision-making.

### 3.5 Network meta-analysis

#### 3.5.1 Network relationships

This study included a total of 17 randomized controlled trials involving 4,220 participants and conducted a network meta-analysis. For outcomes related to depressive symptoms, a total of 10 studies were included (2 RT + AE, 1 Yoga, 4 AE, 1 TCC, and 2 HIIT); for outcomes related to anxiety symptoms, a total of 10 studies were included (2 RT + AE, 2 Yoga, 4 AE, and 2 HIIT); for outcomes related to stress symptoms, a total of 6 studies were included (1 RT + AE, 3 Yoga, 1 AE, 1 TCC). We constructed network evidence maps for the moderating variables of each outcome measure, where each node represents a different type of intervention characteristic (type, frequency, intensity, duration), the size of the node represents the sample size of that intervention, and the lines connecting nodes indicate direct comparisons between two interventions, with the width of the line indicating the number of studies conducting the comparison. Because there are no closed loops in the network of relationships among the various interventions, it is not possible to directly assess the consistency between direct and indirect comparisons using a loop inconsistency test. However, the absence of closed loops does not rule out potential issues with the assumptions of transitivity and consistency underlying indirect comparisons. The validity of indirect comparisons depends on the comparability of clinical and methodological characteristics across studies; if included studies exhibit significant differences in participant characteristics, intervention implementation, or outcome measures, the results of indirect comparisons may be biased. Furthermore, due to the limited number of randomized controlled trials included, the lack of direct comparisons between some intervention nodes, and the low network connectivity density, indirect comparisons rely heavily on assumptions of similarity. More high-quality randomized controlled trials with direct comparisons are needed to validate the current findings and improve the stability of network comparisons. See Appendix 7, Figures 7–10, for a network diagram illustrating the types, frequency, duration, and intensity of physical activity.

#### 3.5.2 Results of the network meta-analysis

The results of the network meta-analysis show that a total of 10 studies reported the effects of exercise interventions on improving depressive symptoms in adolescents. Except for RT + AE, Yoga, AE, TCC, and HIIT all had a positive effect on depressive symptoms; however, only AE and TCC were significantly superior to the control groups. Ten and six studies, respectively, reported on the effects of exercise interventions on anxiety and stress symptoms in adolescents. The results showed that RT + AE, Yoga, AE, and HIIT all had an ameliorative effect on anxiety symptoms, but there were no significant differences compared to the control groups; RT + AE, Yoga, AE, and TCC all had an ameliorative effect on stress symptoms, but similarly, these differences were not statistically significant. Forest plots comparing the effects of different exercise modalities with the control group and a summary table of the effects of different types of exercise interventions are shown in Appendix 7, Figures 11, 12.

#### 3.5.3 Best probability sorting results

According to the best-probability ranking results from SUCRA, regarding exercise types, the intervention effects on adolescent depressive symptoms were, in descending order: TCC (89.77%) > AE (77.10%) > HIIT (55.91%) > Yoga (40.2%) > Control (18.59%) > RE + AE (18.39%); For anxiety symptoms in adolescents, the intervention effectiveness is HIIT (77.47%) > Yoga (62.67%) > AE (61.63%) > RE + AE (34.92%) > Control (13.29%); The intervention effects on adolescents’ stress symptoms were: Yoga (66.42%) > AE (58.95%) > RE + AE (56.61%) > TCC (53.68%) > Control (14.31%). See Appendix 7, Figure 13 for the cumulative probability distribution of exercise types associated with depression, anxiety, and stress.

Regarding exercise frequency, the intervention effects on adolescents’ depressive symptoms were, in descending order: <3 times (87.79%), ≥3 times (57.44%), and Control (4.77%); for anxiety symptoms, the effects were: <3 times (80.39%), ≥3 times (67.14%), and Control (2.46%); the intervention effects on adolescents’ stress symptoms were <3 times (99.49%) superior to the Control group (0.50%). See Appendix 7, Figure 14 for the cumulative probability distribution of exercise frequency by depression, anxiety, and stress.

In terms of exercise duration, the intervention effectiveness for adolescent depressive symptoms was, in descending order: >60 min (69.96%), ≤30 min (60.82%), 30–60 min (55.59%), and the control group (13.61%); For adolescent anxiety symptoms, the intervention effectiveness was as follows: >60 min (91.67%), 30–60 min (58.57%), ≤30 min (39.02%), and Control (10.72%); The intervention effects on adolescents’ stress symptoms were as follows: 30–60 min (77.58%), >60 min (59.93%), ≤30 min (54.88%), and Control (7.6%). See Appendix 7, Figure 15 for the probability plot of cumulative exercise duration for depression, anxiety, and stress.

In terms of exercise intensity, the intervention effects on adolescents’ depressive symptoms were, in descending order: low intensity (91.87%), moderate-to-high intensity (52.29%), and Control (5.83%); for anxiety symptoms, the effects were: moderate-to-high intensity (82.84%), low intensity (64.88%), and Control (2.27%); the intervention effects on adolescents’ stress symptoms were, in descending order, low intensity (80.98%), moderate-to-high intensity (62.51%), and Control (6.15%). See Appendix 7, Figure 16 for the probability plot of cumulative exercise intensity rankings for depression, anxiety, and stress.

It is important to note that the SUCRA ranking results only reflect the probability of different intervention characteristics ranking highest; they do not demonstrate statistically significant or clinically meaningful superiority. Therefore, the SUCRA ranking should be viewed solely as exploratory findings regarding exercise interventions for adolescents’ mental health, rather than as an established optimal exercise prescription.

### 3.6 Risk of publication bias

This study used funnel plots with contour plots and Egger’s asymmetry test to assess publication bias; a *p*-value greater than 0.05 indicates no risk of publication bias (Lin and Chu, 2018; Peters et al., 2008). The results of the Egger’s test showed no statistically significant evidence of funnel plot asymmetry (Depression [*p* = 0.085], Anxiety [*p* = 0.477], and Stress [*p* = 0.362]). However, due to the small number of included studies, the statistical power of this test is limited; therefore, the current results cannot serve as sufficient evidence to rule out publication bias. At the same time, issues such as the small-sample effect, heterogeneity among studies, and selective reporting of outcomes may also lead to funnel plot asymmetry; however, it is difficult to adequately distinguish these factors given the current number of studies. To more comprehensively assess publication bias, we conducted a trim-and-fill analysis using a random-effects model. The results showed that after adjusting for publication bias via the trim-and-fill method, the effect sizes decreased slightly, suggesting that the meta-analysis results are relatively robust. The publication bias funnel plot are provided in Appendix 8.

## 4 Discussion

### 4.1 Summary of finding

Compared with most studies that focus on a single outcome measure (Banyard et al., 2025; Yu et al., 2022), This study also examined the effects of exercise interventions on depression, anxiety, and stress in adolescents, seeking to identify the optimal exercise patterns (e.g., type, frequency) and dosage (e.g., duration, intensity) for improving adolescents’ mental health, and presented the findings in a visual format. Our findings indicate that the various exercise intervention programs included in the study produced beneficial improvements in adolescents’ symptoms of depression, anxiety, and stress, and that regular physical exercise promotes the development of adolescents’ mental health. Differences in the degree of improvement were primarily attributed to moderating variables such as exercise type, frequency, duration, and intensity. According to the independent SUCRA ranking, low-intensity TCC exercises performed less than 3 times per week for more than 60 min may offer potential benefits for adolescents’ depressive symptoms; moderate-to-high-intensity HIIT exercises performed less than 3 times per week for more than 60 min may offer potential benefits for adolescents’ anxiety symptoms; low-intensity yoga sessions performed fewer than 3 times per week for 30–60 min may offer potential benefits for adolescents’ stress symptoms. However, the aforementioned combinations have not yet been directly evaluated in included studies. Future research should conduct more experimental studies with single-arm or multi-arm designs to explore and validate the effectiveness of different intervention characteristics for adolescent mental health interventions. The research summary chart are provided in Appendix 9.

### 4.2 Effect of exercise on adolescent mental health

Physical exercise is not merely a sporting activity but a powerful health intervention that profoundly and comprehensively impacts adolescents’ mental well-being. During physical activity, the simultaneous activation and synergistic interaction of neurochemical, neuroendocrine, neuroplastic, and autonomic nervous system pathways trigger a series of complex and positive changes in both the brain and body (Liu and Wang, 2021), thereby effectively enhancing adolescents’ psychological health.

From a physiological perspective, the onset of depression is often associated with impaired function of monoamine neurotransmitters in the human body. Neurotransmitters such as serotonin, norepinephrine, and dopamine regulate an individual’s mood, sense of pleasure, and motivation. Physical exercise can significantly elevate levels of these neurotransmitters; for example, brain-derived neurotrophic factor (BDNF) increases following exercise (Kramer et al., 2023). Research indicates that alterations in depression and stress responses can be measured through cortisol levels and systemic inflammation (Yates, 2025; Zhou and Fabiano, 2024). Furthermore, it has been clearly demonstrated that exercise not only increases hippocampal and prefrontal cortex volume, thereby promoting the restoration of common neurobiological mechanisms and alleviating depressive symptoms in some patients (Pereira et al., 2021), but also ensures that the benefits of exercise persist after the intervention concludes. The physiological core of anxiety lies in the excessive excitation and dysregulation of the sympathetic nervous system. Compared to depression, individuals with anxiety disorders typically exhibit a deficiency in gamma-aminobutyric acid (GABA) (Garvey et al., 2023). As the primary inhibitory neurotransmitter in the brain, GABA primarily functions to reduce neuronal excitability and produce anxiolytic effects. Research has clearly demonstrated that exercise enhances GABA synthesis and release (Cao et al., 2022), thereby alleviating anxiety symptoms in individuals. Prolonged psychological stress can disrupt the hypothalamic–pituitary–adrenal (HPA) axis, leading to persistently elevated cortisol levels that may cause damage to the brain and hippocampus. The core stress-relieving effect of exercise lies in its ability to optimize HPA axis function and enhance prefrontal cortex regulation, thereby improving stress coping efficiency and strengthening psychological resilience (Smith et al., 2025).

Compared to physiological mechanisms that focus on internal bodily chemical reactions, psychological mechanisms emphasize exercise’s role in enhancing individuals’ emotional, cognitive, and social well-being. Psychological research suggests that physical activity can be viewed as a psychological intervention that activates behavior and reshapes cognition. It builds psychological resilience in adolescents through multiple dimensions, including creating social opportunities, enhancing a sense of control, and fostering experiences of success (Granero-Jiménez et al., 2022; Knapp et al., 2023). Firstly, group-based sports interventions provide adolescents with social support, peer connections, and a sense of belonging—elements crucial for alleviating loneliness and building social support networks (Anderson et al., 2023). Relevant sports psychology assessments indicate that sustained participation in team activities during the transition from adolescence to young adulthood promotes better mental health (Murray et al., 2021). Secondly, improvements in physical fitness and the achievement of athletic goals can significantly enhance adolescents’ sense of self-efficacy and mastery, which can be generalized to other areas of life, such as academics, thereby contributing to an improved quality of life (Fossati et al., 2021). Finally, regular exercise not only helps build a healthy physique, improve adolescents’ body image, and enhance their satisfaction with their own bodies, but also helps distract them from daily worries and negative emotions (Guo and Zhang, 2022), alleviating psychological stress in everyday life.

It is worth noting that this review aims to evaluate the effects of exercise interventions on outcomes related to depression, anxiety, and stress, and to compare the relative rankings of different types, frequencies, durations, and intensities of exercise. The included studies all used psychological scales to assess outcomes and did not directly measure neurobiological markers (such as brain-derived neurotrophic factor, hippocampal volume, inflammatory markers, hypothalamic–pituitary–adrenal axis function, or monoamine neurotransmitters). Therefore, this review cannot directly validate any neurobiological mechanisms; the above discussions regarding mechanisms are based on hypotheses proposed in the prior literature and constitute interpretive speculation rather than direct findings of this review.

### 4.3 Comparison and integration with previous research

Scholars have offered various perspectives on the optimal model and dosage of exercise interventions for adolescent mental health. Regarding the consistency of research findings, some studies indicate that individuals who exercise less than 3 h per week have a higher risk of developing depression than those who exercise 5 h or more per week (Kim, 2022). Resistance training can lead to mild to moderate improvements in patients’ anxiety symptoms (Herring and Meyer, 2024). Based on an examination of the specific molecular mechanisms underlying the antidepressant effects of exercise, Ren concludes that engaging in moderate-to-vigorous aerobic exercise 3–5 times per week can effectively alleviate depressive symptoms (Ren and Xiao, 2023). Other studies suggest that resistance training is more effective in alleviating depression than anxiety, which is consistent with our findings that conventional exercise interventions may be more effective in reducing anxiety symptoms in adolescents (Herring and Meyer, 2024). Since the synthesis of GABA requires a certain level of exercise intensity, conventional exercise interventions are most appropriate (Cao et al., 2022). Our findings are consistent with the above views, and by further refining these findings, we provide more specific guidelines for exercise interventions.

However, there remain some discrepancies between our findings and those of previous studies regarding the optimal type and intensity of exercise interventions for mental health. Concerning the optimal type of exercise, some studies suggest that yoga does not have a significant effect on depression in adolescents (Wang et al., 2022). Vigorous exercise is the most effective intensity for treating depression, which differs from our TCC study’s findings, which indicate that our intervention may yield better results for depressive symptoms (Li et al., 2025). Regarding exercise dosage, some studies have indicated that the optimal dose for alleviating depressive symptoms is a single session of 30–45 min of moderate-intensity exercise, which yields the best results for treating depression (Wang et al., 2025). This differs from our findings, which show that a single exercise session lasting more than 60 min also yields good results for treating depression. A review of the relevant original literature suggests that these discrepancies may stem from differences in the age of the study populations and sample sizes. Despite these inconsistencies in research findings, this study builds upon previous research to provide new insights and empirical evidence, which can serve as a valuable reference for the sustainable development of non-pharmacological interventions for adolescent mental health.

### 4.4 Potential sources of heterogeneity and limitations on the interpretability of subgroup analysis results

The results showed significant heterogeneity in the analyses of anxiety (*I*^2^ = 84.7%) and depression (*I*^2^ = 58.0%). This heterogeneity may stem from multiple factors, including differences in participant characteristics, intervention characteristics, outcome assessment tools, and study methodology; while imbalances in the number of studies and sample sizes across subgroups may have further amplified the heterogeneity, they are not the sole or primary explanation. Regarding participant characteristics, differences in the baseline characteristics of the populations included in different studies may lead to varying responses to the intervention, specifically including factors such as age, gender ratio, severity of baseline symptoms, and clinical diagnostic criteria; Regarding intervention characteristics, even for interventions involving the same type of exercise, differences in implementation details may lead to varying effect sizes. These include factors such as exercise type, frequency, duration, intensity, whether the exercise is supervised, whether it is conducted in a group setting, and adherence to the intervention; regarding assessment tools, differences in the sensitivity and measurement dimensions of various tools may introduce heterogeneity. For example, factors such as the cutoff values of assessment tools, the timing of assessments, and whether an intention-to-treat analysis is used may all contribute to the emergence of heterogeneity; With regard to methodological differences, factors such as randomization methods, concealed allocation, blinding, completeness of outcome reporting, sample size calculation, and statistical methods may all influence the estimation of effect sizes, thereby leading to high heterogeneity. Furthermore, some subgroups are based on only 1–2 studies, limiting their interpretability; their results cannot be used to infer true differences between subgroups and should not serve as the basis for clinical recommendations. Imbalances in the number of studies and sample sizes across subgroups may further exacerbate heterogeneity, but are not sufficient on their own to explain its entirety.

### 4.5 Future research directions

Although the positive effects of exercise on adolescent mental health have been well-documented, several potential research directions warrant further exploration. First, the dose–response relationship of mental health interventions for individuals with chronic conditions such as diabetes and hypertension remains to be investigated (Roberts et al., 2021). Given their physical limitations, exercise intervention programs for these populations must be designed with safety and feasibility as paramount considerations. Second, there remains room for expansion in the types of exercise interventions. Current research on mind–body exercises and emerging exercise interventions for adolescent mental health is insufficient. Future studies should focus on enhancing the diversity of exercise intervention types, such as further investigating the impact of exercise snacks (Jones et al., 2024; Weston et al., 2025), fitness qigong, and Pilates on the mental health of various populations. Third, given the gender differences in physical exercise capacity, it remains to be explored how to accurately determine the dose–response relationship of exercise intervention protocols for different genders during physical activity while ensuring intervention efficacy. Additionally, research topics such as exercise effects at different time intervals, the sustainability of intervention outcomes, technology-assisted exercise interventions, and the development of the exercise intervention mental health industry all warrant attention.

### 4.6 Potential limitations

Although this study effectively evaluated and explored the effects of exercise interventions on adolescents’ mental health, there are still some notable potential limitations: (1) This study included only published English-language studies and did not include non-English literature or manuscripts currently in the publication process, which may lead to potential language bias and affect the comprehensiveness of the study selection. (2) Limiting the control group to “no exercise intervention” may have excluded high-quality randomized controlled trials that employed active control groups (e.g., health education, attention control). (3) All included original studies were randomized controlled trials; however, implementing blinding for participants and researchers during the experimental process is often difficult, which may introduce a risk of bias. (4) Significant heterogeneity (*I^2^* > 50%) persists among the studies, which may be attributed to differences in exercise protocols, measurement tools (various test scales), and baseline participant characteristics. Although this study conducted subgroup and sensitivity analyses to address this issue, the exact cause of the significant heterogeneity remains unclear. (5) Although NMA allows for the simultaneous comparison of multiple exercise interventions within the same evidence network, its results rely on the transitivity assumption—that is, the assumption that effect moderators are comparable across studies. If this assumption does not hold, the results of indirect comparisons may be biased. Furthermore, the number of included studies, sample sizes, and the number of events may limit the robustness of network connections; some comparisons may be based solely on indirect evidence and should be interpreted with caution. (6) The SUCRA value reflects only the probability that an intervention is the best option; it is not a direct measure of effect size. Furthermore, it is sensitive to network structure, the number of included studies, and the uncertainty in effect estimates. The SUCRA difference between higher-ranked and lower-ranked interventions may not be statistically significant. Therefore, SUCRA rankings should be interpreted in conjunction with effect estimates and their confidence intervals; the superiority or inferiority of an intervention should not be inferred based solely on its ranking. (7) The dose parameters (e.g., frequency, intensity, duration) of exercise interventions in this study were primarily derived from post-hoc categorizations or independent analyses of the original studies, rather than from specifically designed dose–response trials. The operational definitions of “dose” vary across studies, and intervention components are often implemented in combination, making it difficult to precisely isolate the independent contributions of each component through an NMA or meta-regression. Therefore, the current analysis provides a comparison of relative intervention strategies rather than precise recommendations for optimal dosing.

## 5 Conclusion

This network meta-analysis synthesized existing evidence from previous studies and provided researchers and clinical professionals with several key findings regarding the effects of physical activity on improving adolescents’ mental health. The results indicate that physical activity interventions, as a non-pharmacological treatment, play a positive role in improving adolescents’ mental health, and identified potential exercise patterns and dosages that may yield better outcomes based on the SUCRA ranking. However, given the limited number of studies, moderate overall methodological quality, publication bias, and heterogeneity among studies, these conclusions should be interpreted with caution and require further validation. Overall, the optimal exercise patterns and dosages identified in this study are based on exploratory findings from the existing evidence; future research is needed to further examine the effects of interventions across different variables.

## Data Availability

The original contributions presented in the study are included in the article/Supplementary material, further inquiries can be directed to the corresponding author.
