# Supplementary material for: Modalities and dosage of exercise interventions for improving adolescent mental health: a systematic review and meta-analysis
Source: Front Psychol. 2026 Oct 28;17:1986368. doi: 10.3389/fpsyg.2026.1986368 (PMC13751402; doi:10.3389/fpsyg.2026.1986368)
Supplement: Supplementary file 1 [file Supplementary_file_1.docx]

**Optimal model and dosage of exercise interventions to improve adolescent mental health: systematic review and network meta-analysis of randomized controlled trials**

**Supplemental files**

**Contents**

[Appendix 1 Search strategy	1](#_Toc2044)

[Table 1 Search strategy in Database	1](#_Toc2063)

[Appendix 2 Complete eligibility criteria for systematic review	3](#_Toc8536)

[Table 2 Complete eligibility criteria for systematic review.	3](#_Toc2341)

[Appendix 3 Search Results	4](#_Toc30258)

[Fig. 1 PRISMA flow diagram for included and excluded studies.	4](#_Toc17246)

[Appendix 4 Study characteristics	5](#_Toc22995)

[Table 3 The characteristics for the studies included.	5](#_Toc11189)

[Appendix 5 Risk of Bias and Quality Assessment	6](#_Toc2234)

[Fig. 2 Risk of bias graph and risk summary graph for included literature.	6](#_Toc605)

[Table 4 Methodological quality assessment (PEDro)	6](#_Toc2386)

[Appendix 6 Pairwise meta-analysis results	7](#_Toc26377)

[Fig. 3 Primary pooled effect sizes for the outcomes.	7](#_Toc19344)

[Fig. 4 A sensitivity analysis based on leave-one-out.	7](#_Toc25768)

[Fig. 5 Subgroup analysis based on depression.	8](#_Toc17996)

[Fig. 6 Subgroup analysis based on anxiety.	8](#_Toc22471)

[Appendix 7 Pairwise meta-analysis results	9](#_Toc2194)

[Fig. 7 Evidence maps of the type networks for (A) depression, (B) anxiety, and (C) stress.	9](#_Toc27955)

[Fig. 8 Evidence maps of the frequency networks for (A) depression, (B) anxiety, and (C) stress.	9](#_Toc9784)

[Fig. 9 Evidence maps of the temporal networks for (A) depression, (B) anxiety, and (C) stress.	9](#_Toc8859)

[Fig. 10 Evidence map of exercise intensity for (A) depression, (C) anxiety, and (D) stress.	10](#_Toc25222)

[Fig. 11 Forest plots of the effects of different exercises compared to the control group.	10](#_Toc16699)

[Fig. 12 A league chart showing the types of exercise associated with (A) depression, (B) anxiety, and (C) stress.	11](#_Toc20654)

[Fig. 13 Cumulative ranking probability plots of Sport Type for (A) depression, (B) anxiety, and (C) stress.	11](#_Toc19154)

[Fig. 14 Cumulative ranking probability plots of exercise frequency for (A) depression, (B) anxiety, and (C) stress.	12](#_Toc8543)

[Fig. 15 Cumulative ranking probability plots of exercise duration for (A) depression, (B) anxiety, and (C) stress.	12](#_Toc21372)

[Fig. 16 Cumulative ranking probability plots of exercise intensity for (A) depression, (B) anxiety, and (C) stress.	13](#_Toc19913)

[Appendix 8 Funnel plot of included studies	14](#_Toc22309)

[Fig. 17 Funnel plot of included studies.	14](#_Toc5052)

[Appendix 9 Funnel plot of included studies	15](#_Toc20389)

[Fig. 18 The Summary of the article.	15](#_Toc5071)

**Appendix 1 Search strategy**

Table 1 Search strategy in Database

| Database | Search strategy |
| --- | --- |
| Web of Science | A=(adolescent or teenager or youth or juveniles) and (exercise or physical exercise or physical activity or sports intervention or exercise intervention) and (mental health or psychological health or psychological well-being) and (despondent or clinical depression or depression or depressive disorder or depressive illness) and (neurosis or nephropathy or anxiety disorder or anxiety) and (stress or strain or stress symptoms)(Title)  B=(adolescent or teenager or youth or juveniles) and (exercise or physical exercise or physical activity or sports intervention or exercise intervention) and (mental health or psychological health or psychological well-being) and (despondent or clinical depression or depression or depressive disorder or depressive illness) and (neurosis or nephropathy or anxiety disorder or anxiety) and (stress or strain or stress symptoms)(Abstract)  “A” OR “B” |
| PubMed | (adolescent[Title/Abstract] OR teenager[Title/Abstract] OR youth[Title/Abstract] OR juveniles[Title/Abstract]) AND (exercise[Title/Abstract] OR physical exercise[Title/Abstract] OR physical activity[Title/Abstract] OR sports intervention[Title/Abstract] OR exercise intervention[Title/Abstract]) AND (mental health[Title/Abstract] OR psychological health[Title/Abstract] OR psychological well-being[Title/Abstract]) AND (despondent[Title/Abstract] OR clinical depression[Title/Abstract] OR depression[Title/Abstract] OR depressive disorder[Title/Abstract] OR depressive illness[Title/Abstract]) AND (neurosis[Title/Abstract] OR nephropathy[Title/Abstract] OR anxiety disorder[Title/Abstract] OR anxiety[Title/Abstract]) AND (stress[Title/Abstract] OR strain[Title/Abstract] OR stress symptoms[Title/Abstract]) |
| Embase | (adolescent OR teenager OR youth OR juveniles) in Title Abstract Keyword AND (exercise OR physical exercise OR physical activity OR sports intervention OR exercise intervention) in Title Abstract Keyword AND (mental health OR psychological health OR psychological well-being) in Title Abstract Keyword AND (despondent OR clinical depression OR depression OR depressive disorder OR depressive illness) in Title Abstract Keyword AND (neurosis OR nephropathy OR anxiety disorder OR anxiety) in Title Abstract Keyword AND (stress OR strain OR stress symptoms) in Title Abstract Keyword |
| The Cochrane Library | (adolescent OR teenager OR youth OR juveniles) in Title Abstract Keyword AND (exercise OR physical exercise OR physical activity OR sports intervention OR exercise intervention) in Title Abstract Keyword AND (mental health OR psychological health OR psychological well-being) in Title Abstract Keyword AND (despondent OR clinical depression OR depression OR depressive disorder OR depressive illness) in Title Abstract Keyword AND (neurosis OR nephropathy OR anxiety disorder OR anxiety) in Title Abstract Keyword AND (stress OR strain OR stress symptoms) in Title Abstract Keyword |
| SportDiscus | A=(adolescent or teenager or youth or juveniles) and (exercise or physical exercise or physical activity or sports intervention or exercise intervention) and (mental health or psychological health or psychological well-being) and (despondent or clinical depression or depression or depressive disorder or depressive illness) and (neurosis or nephropathy or anxiety disorder or anxiety) and (stress or strain or stress symptoms)(Title)  B=(adolescent or teenager or youth or juveniles) and (exercise or physical exercise or physical activity or sports intervention or exercise intervention) and (mental health or psychological health or psychological well-being) and (despondent or clinical depression or depression or depressive disorder or depressive illness) and (neurosis or nephropathy or anxiety disorder or anxiety) and (stress or strain or stress symptoms)(Abstract)  “A” OR “B” |
| Scopus | (adolescent OR teenager OR youth OR juveniles) in Title Abstract Keyword AND (exercise OR physical exercise OR physical activity OR sports intervention OR exercise intervention) in Title Abstract Keyword AND (mental health OR psychological health OR psychological well-being) in Title Abstract Keyword AND (despondent OR clinical depression OR depression OR depressive disorder OR depressive illness) in Title Abstract Keyword AND (neurosis OR nephropathy OR anxiety disorder OR anxiety) in Title Abstract Keyword AND (stress OR strain OR stress symptoms) in Title Abstract Keyword |

**Appendix 2 Complete eligibility criteria for systematic review**

Table 2 Complete eligibility criteria for systematic review.

**Inclusion criteria**

P(Population): An adolescent population (10-19 years old) that meets the definition of the World Health Organization.

I(Intervention): The experimental group received various forms of exercise intervention, and if there were multiple experimental groups in the study, only the exercise intervention group was included.

C(Control): Control group without exercise intervention.

O(Outcome): Relevant scales of internationally recognized measures of mental health were used and their scores were used as study outcomes.

S(Study design): Randomized controlled trial (RCT) of exercise interventions for adolescent mental health.

**Exclusion criteria**

1. Non-randomized controlled trials, non-English literature.

**2.** Reviews, conferences, and repeat publications.

**3.** Experimental data is missing, and attempts to obtain the complete data by contacting the author have taken over a week.

**4.** Unexplored intervention effects of exercise interventions on adolescent mental health.

**5.** Literature that did not meet any of the inclusion criteria.

**Appendix 3 Search Results**

Fig. 1 PRISMA flow diagram for included and excluded studies.

**Appendix 4 Study characteristics**

Table 3 The characteristics for the studies included.

| **Study** | **Participants** | **Intervention characteristics** | | | | | **Outcomes** | **PEDro** |
| --- | --- | --- | --- | --- | --- | --- | --- | --- |
|  |  | **Intensity** | **Duration** | **Time** | **Frequency** | **Type** |  |  |
| Arnaud et al.  (2022) | Sample size(T/C):20/20  Gender(M/F):15/25  Age:15.35±0.15 | 40-59%HRR | 5 | 60 | 4 | RT、AE | HAMD、  STAI | 9 |
| Bethany et al.  (2017) | Sample size(T/C):114/91  Gender(M/F):75/130  Age:12.64 | Low-intensity yoga training | 16 | 35 | 2 | Yoga | BRUMS、PSS | 7 |
| Chang Liu et al.  (2025) | Sample size(T/C):40/40  Gender(M/F):72/8  Age:14.74±1.59 | Low to moderate intensity, gradually increasing | 12 | 60 | 1 | AE | DASS-21 | 8 |
| Harish et al.  (2023) | Sample size(T/C):996/1004  Gender(M/F):——  Age:13-15 | Low-intensity yoga training | 17 | 45 | 1 | Yoga | ADOSS | 7 |
| Heidrun et al.  (2018) | Sample size(T/C):21/23  Gender(M/F):11/33  Age:15.9±1.1 | Low-intensity whole-body vibration training | 6 | 30 | 4 | AE | DIKJ | 7 |
| Jadson et al.  (2025) | Sample size(T/C):165/141  Gender(M/F):140/166  Age:13.6±0.72 | Borg’s rating of perceived  exertion scale | 12 | 20 | 2 | RT、AE | DASS-21 | 6 |
| Jiayuan Zhang et al.(2018) | Sample size(T/C):32/32  Gender(M/F):23/41  Age:18.41±2.01 | Low-intensity Tai Chi training | 8 | 90 | 2 | TCC | PHQ-9、PSS | 8 |
| Jordan et al.  (2025A) | Sample size(T/C):12/13  Gender(M/F):16/21  Age:16.1 ± 0.2 | ＜64%HRmax | 6 | 20 | 2 | Yoga | PSS | 8 |
| Jordan et al.  (2025B) | Sample size(T/C):12/13  Gender(M/F):16/21  Age:16.1 ± 0.2 | ＜64%HRmax | 6 | 20 | 2 | RT、AE | PSS | 8 |
| Niranjan et al.  (2022) | Sample size(T/C):45/44  Gender(M/F):0/89  Age:12.78 ± 0.73 | Low-intensity yoga training | 8 | 60 | 4 | Yoga | STAI | 7 |
| Qian Gu et al.  (2024A) | Sample size(T/C):33/33  Gender(M/F):0/95  Age:16.50± 0.31 | 40-59%HRR | 8 | 40 | 3 | AE | BDI-13 | 8 |
| Qian Gu et al.  (2024B) | Sample size(T/C):29/33  Gender(M/F):0/95  Age:16.65± 0.44 | 40-59%HRR | 8 | 40 | 3 | AE | BDI-13 | 8 |
| Wu J et al.  (2025A) | Sample size(T/C):50/50  Gender(M/F):——  Age:15.5 | Low-intensity sports games | 6 | 60 | 2 | AE | FCV-19 | 4 |
| Wu J et al.  (2025B) | Sample size(T/C):50/50  Gender(M/F):——  Age:15.5 | Low-intensity sports games | 6 | 60 | 2 | AE | FCV-19 | 4 |
| Yingfeng Zheng et al. (2021) | Sample size(T/C):485/469  Gender(M/F):499/455  Age:13.5± 0.5 | Low-intensity exercise intervention | 2 | 15 | 5 | AE | SCAS | 8 |
| Yutong Li et al.  (2025A) | Sample size(T/C):15/15  Gender(M/F):30/0  Age:17.1± 0.7 | 85-95%HRmax | 8 | 62 | 5 | HIIT | MSPYA | 4 |
| Yutong Li et al.  (2025B) | Sample size(T/C):15/15  Gender(M/F):0/30  Age:17.1± 0.7 | 85-95%HRmax | 8 | 62 | 5 | HIIT | MSPYA | 4 |

Abbreviations: **HRmax**=Maximum heart rate; **HRR**=Heart Rate Reserve; **HIIT**=high intensity interval training; **RT**=resistance training; **AE**=aerobic exercise; **TCC**=Tai Chi Chuan; **CDI**=Children’s Depression Inventory; **STAI**=State-Trait Anxiety Inventory; **BRUMS**=The Brunel University Mood Scale; **PSS**=Perceived Stress Scale; **DASS-21**=Depression, Anxiety, and Stress Scale - Short Form; **ADOSS**=ADOlescence Stress Scale; **DIKJ**=Depressionsinventar für Kinder und Jugendliche; **PHQ-9**=Nine-item patient health questionnaire depression scale; **BDI-13**=13-item Beck Depression Inventory short form; **CES-D**=The Center for Epidemiological Studies Depression Scale; **FCV-19**=Fear of COVID-19; **SCAS**=Spence Children’s Anxiety Scale; **MSPYA**=Multidimensional Scale of Psychological States in Adolescents.

**Appendix 5 Risk of Bias and Quality Assessment**

Fig. 2 Risk of bias graph and risk summary graph for included literature.

Table 4 Methodological quality assessment (PEDro)

| **Study, year** | **D1** | **D2** | **D3** | **D4** | **D5** | **D6** | **D7** | **D8** | **D9** | **D10** | **D11** | **Total** |
| --- | --- | --- | --- | --- | --- | --- | --- | --- | --- | --- | --- | --- |
| Arnaud et al. 2022 | Y | 1 | 1 | 1 | 1 | 1 | 1 | 1 | 0 | 1 | 1 | 9 |
| Bethany et al. 2017 | Y | 1 | 1 | 1 | 0 | 0 | 0 | 1 | 1 | 1 | 1 | 7 |
| Chang Liu et al. 2025 | Y | 1 | 1 | 1 | 1 | 1 | 1 | 0 | 0 | 1 | 1 | 8 |
| Harish et al. 2023 | Y | 1 | 1 | 1 | 0 | 0 | 0 | 1 | 1 | 1 | 1 | 7 |
| Heidrun et al. 2018 | Y | 1 | 1 | 1 | 0 | 0 | 0 | 1 | 1 | 1 | 1 | 7 |
| Jadson et al. 2025 | Y | 1 | 0 | 1 | 0 | 0 | 0 | 1 | 1 | 1 | 1 | 6 |
| Jiayuan Zhang et al. 2018 | Y | 1 | 1 | 0 | 1 | 0 | 1 | 1 | 1 | 1 | 1 | 8 |
| Jordan et al. 2025A | Y | 1 | 1 | 1 | 0 | 0 | 1 | 1 | 1 | 1 | 1 | 8 |
| Jordan et al. 2025B | Y | 1 | 1 | 1 | 0 | 0 | 1 | 1 | 1 | 1 | 1 | 8 |
| Niranjan et al. 2022 | Y | 1 | 0 | 1 | 0 | 0 | 1 | 1 | 1 | 1 | 1 | 7 |
| Qian Gu et al. 2024A | Y | 1 | 1 | 1 | 1 | 0 | 0 | 1 | 1 | 1 | 1 | 8 |
| Qian Gu et al. 2024B | Y | 1 | 1 | 1 | 1 | 0 | 0 | 1 | 1 | 1 | 1 | 8 |
| Wu J et al. 2025A | N | 1 | 0 | 0 | 0 | 0 | 0 | 1 | 0 | 1 | 1 | 4 |
| Wu J et al. 2025B | N | 1 | 0 | 0 | 0 | 0 | 0 | 1 | 0 | 1 | 1 | 4 |
| Yingfeng Zheng et al. 2021 | Y | 1 | 1 | 1 | 0 | 0 | 1 | 1 | 1 | 1 | 1 | 8 |
| Yutong Li et al. 2025A | Y | 1 | 0 | 0 | 0 | 0 | 0 | 1 | 0 | 1 | 1 | 4 |
| Yutong Li et al. 2025B | Y | 1 | 0 | 0 | 0 | 0 | 0 | 1 | 0 | 1 | 1 | 4 |

Note: studies scoring＞7are considered high quality, those scoring 5-7 are considered **moderate quality**, and those scoring ≤4 are considered **low quality**.

**Appendix 6 Pairwise meta-analysis results**

Fig. 3 Primary pooled effect sizes for the outcomes.

**Note:** K: the total number of effects included in the pooled effect size;Experiment: the corresponding values in this column are the sample sizes for the experimental group; Control: the corresponding values in this column are the sample sizes of the control group; Hedge’s g: the effect size indicators used in the pooled; 95%CI = 95% confidence interval; p-value: statistically significant p values for pooled results; *I*^2^ : quantitative indicators of heterogeneity.

Fig. 4 A sensitivity analysis based on leave-one-out.

Fig. 5 Subgroup analysis based on depression.

Fig. 6 Subgroup analysis based on anxiety.

**Appendix 7 Pairwise meta-analysis results**

Fig. 7 Evidence maps of the type networks for (A) depression, (B) anxiety, and (C) stress.

Fig. 8 Evidence maps of the frequency networks for (A) depression, (B) anxiety, and (C) stress.

Fig. 9 Evidence maps of the temporal networks for (A) depression, (B) anxiety, and (C) stress.

Fig. 10 Evidence map of exercise intensity for (A) depression, (C) anxiety, and (D) stress.

Fig. 11 Forest plots of the effects of different exercises compared to the control group.

Fig. 12 A league chart showing the types of exercise associated with (A) depression, (B) anxiety, and (C) stress.

Fig. 13 Cumulative ranking probability plots of Sport Type for (A) depression, (B) anxiety, and (C) stress.

Fig. 14 Cumulative ranking probability plots of exercise frequency for (A) depression, (B) anxiety, and (C) stress.

Fig. 15 Cumulative ranking probability plots of exercise duration for (A) depression, (B) anxiety, and (C) stress.

Fig. 16 Cumulative ranking probability plots of exercise intensity for (A) depression, (B) anxiety, and (C) stress.

**Appendix 8 Funnel plot of included studies**

Fig. 17 Funnel plot of included studies.

**Appendix 9 Funnel plot of included studies**

Fig. 18 The Summary of the article.
